# Next-generation proteasome inhibitor oprozomib synergizes with modulators of the unfolded protein response to suppress hepatocellular carcinoma

**DOI:** 10.18632/oncotarget.9222

**Published:** 2016-05-07

**Authors:** Yves-Paul Vandewynckel, Céline Coucke, Debby Laukens, Lindsey Devisscher, Annelies Paridaens, Eliene Bogaerts, Astrid Vandierendonck, Sarah Raevens, Xavier Verhelst, Christophe Van Steenkiste, Louis Libbrecht, Anja Geerts, Hans Van Vlierberghe

**Affiliations:** ^1^ Department of Hepatology and Gastroenterology, Ghent University, Ghent, Belgium; ^2^ Department of Pathology, Ghent University Hospital, Ghent, Belgium

**Keywords:** hepatocellular carcinoma, endoplasmic reticulum, stress, unfolded protein response, proteasome inhibitor

## Abstract

Hepatocellular carcinoma (HCC) responds poorly to conventional systemic therapies. The first-in-class proteasome inhibitor bortezomib has been approved in clinical use for hematologic malignancies and has shown modest activity in solid tumors, including HCC. However, a considerable proportion of patients fail to respond and experience important adverse events. Recently, the next-generation orally bioavailable irreversible proteasome inhibitor oprozomib was developed. Here, we assessed the efficacy of oprozomib and its effects on the unfolded protein response (UPR), a signaling cascade activated through the ATF6, PERK and IRE1 pathways by accumulation of unfolded proteins in the endoplasmic reticulum, in HCC. The effects of oprozomib and the role of the UPR were evaluated in HCC cell lines and in diethylnitrosamine-induced and xenograft mouse models for HCC. Oprozomib dose-dependently reduced the viability and proliferation of human HCC cells. Unexpectedly, oprozomib-treated cells displayed diminished cytoprotective ATF6-mediated signal transduction as well as unaltered PERK and IRE1 signaling. However, oprozomib increased pro-apoptotic UPR-mediated protein levels by prolonging their half-life, implying that the proteasome acts as a negative UPR regulator. Supplementary boosting of UPR activity synergistically improved the sensitivity to oprozomib via the PERK pathway. Oral oprozomib displayed significant antitumor effects in the orthotopic and xenograft models for HCC, and importantly, combining oprozomib with different UPR activators enhanced the antitumor efficacy by stimulating UPR-induced apoptosis without cumulative toxicity. In conclusion, next-generation proteasome inhibition by oprozomib results in dysregulated UPR activation in HCC. This finding can be exploited to enhance the antitumor efficacy by combining oprozomib with clinically applicable UPR activators.

## INTRODUCTION

Hepatocellular carcinoma (HCC) is the second leading cause of cancer-related mortality worldwide [1]. Resection and transplantation are the only potentially curative treatments available following detection of a small HCC [2]. For the majority of patients with locally advanced disease, however, transarterial embolization and the multi-kinase inhibitor sorafenib are the only approved treatments. Unfortunately, both provide a limited survival benefit [2].

The ubiquitin-proteasome pathway is responsible for the degradation of misfolded proteins as well as short-lived mediators of signaling pathways regulating cell proliferation and survival [3]. Proteasome inhibition leads to the accumulation of these substrates, resulting in concomitant activation of pro- and antiproliferative signals, disruption of cell-cycle regulation, and, ultimately, apoptosis. Bortezomib is the first-in-class proteasome inhibitor clinically used for the treatment of multiple myeloma. Bortezomib-induced cell death is related to induction of endoplasmic reticulum (ER) stress, inhibition of nuclear factor kappa B, activation of caspase-8 and generation of oxidative stress [3, 4]. Multiple clinical trials have demonstrated that this small-molecule possesses antitumor activity in a variety of human cancers [5, 6]. Despite promising preclinical results [5], a multicenter single-arm phase II trial assessing the activity of bortezomib in HCC has demonstrated modest antitumor effects, indicating intrinsic or acquired resistance [4, 7]. In addition, the majority of the patients developed important adverse events, including peripheral neuropathy [7]. Nevertheless, the good clinical outcome of bortezomib in multiple myeloma led to the development of next-generation proteasome inhibitors, such as carfilzomib, that selectively and irreversibly bind to the proteasome in order to enhance its inhibition, improve antitumor activity and decrease toxicity by reducing off-target effects [8, 9]. A phase III trial showed that intravenously administered carfilzomib improved progression-free survival in myeloma with a favorable risk-benefit profile [10]. Recently, an orally bioavailable analogue of carfilzomib, called oprozomib (OZ), was developed [9].

Proteasome inhibition is thought to trigger the accumulation of misfolded proteins in the ER, which activates the unfolded protein response (UPR) [4]. Three major ER stress transducers have been identified: PKR-like endoplasmic reticulum kinase (PERK), inositol-requiring enzyme-1 (IRE1) and activating transcription factor 6 (ATF6) [11]. Following release of chaperone glucose-regulated protein, 78 kDa (GRP78), PERK phosphorylates eukaryotic initiation factor 2α (eIF2α) leading to repression of global translation. However, the phosphorylated form of this factor favors selective translation of activating transcription factor 4 (*ATF4*), which activates genes involved in protein quality control, amino acid biosynthesis as well as apoptosis regulators such as c/EBP-homologous protein (*CHOP*) [11]. Activation of IRE1 results in splicing of unspliced X-box-binding protein 1 (*XBP1u*) mRNA to generate a more active spliced XBP1 (*XBP1s*), which induces genes involved in protein folding such as endoplasmic reticulum DnaJ homolog 4 (*ERDJ4*), protein degradation and redox homeostasis [11]. ATF6 is mobilized to the Golgi where it is cleaved by regulated intramembrane proteolysis (RIP), which involves the site-1 (S1P) and site-2 (S2P) proteases, releasing a transcriptionally active fragment. This pathway induces the expression of chaperones, such as GRP78, glucose-regulated protein, 94 kDa (GRP94), protein disulfide isomerase A4 (PDIA4), calreticulin (CALR) and endoplasmic oxidoreductin-1-like protein (ERO1L), XBP1u and of proteins stimulating protein degradation, such as homocysteine-inducible, ER stress-inducible, ubiquitin-like domain member 1 (HERPUD1) [12].

In this study, we provide a molecular clue to OZ's mode of action and identified the therapeutic potential of OZ in monotherapy and in combination with modulators of the UPR *in vitro* and in mouse models for HCC. Finally, our data illustrate that the proteasome serves a distinct function in restraint of UPR signaling by managing the UPR-induced protein turnover.

## RESULTS

### Supplementary ER stress increases the sensitivity of HCC cells to proteasome inhibition

**Figure 1:**
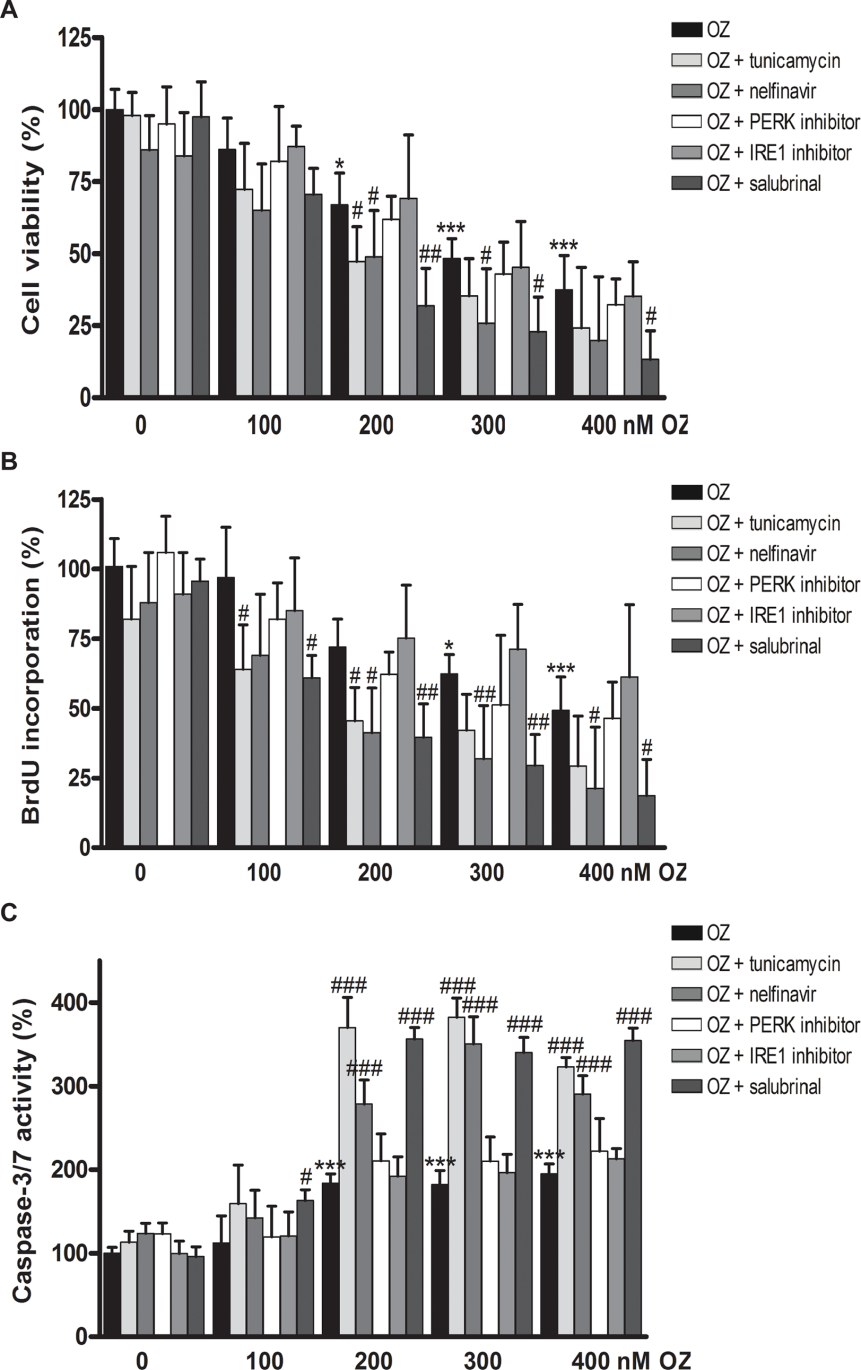
Antiproliferative and pro-apoptotic effects of oprozomib in monotherapy or in combination with modulators of ER stress in human hepatoma HepG2 cells (**A**) MTT assay (**B**) BrdU incorporation (**C**) Caspase-3/7 activity. OZ: oprozomib. *p < 0.05, **p < 0.01, ***p < 0.001 compared to oprozomib 0 nM; ^#^*p* < 0.05, ^##^*p* < 0.01, ^###^*p* < 0.001 compared to the respective concentration of oprozomib alone. Results are representative of 2 independent experiments.

Here, we aim to assess the effect of OZ alone or in combination with UPR modulators on the viability, proliferation and executioner caspase-3/7 activity of HCC cells. Combination with the chemical ER stress inducer tunicamycin, which inhibits N-linked protein glycosylation, or with the recently developed small-molecules selectively inhibiting the IRE1 or PERK pathway or with salubrinal, which inhibits eIF2α dephosphorylation, was evaluated [15]. In HepG2 cells, 48 hours of incubation with 100–400 nM OZ dose-dependently reduced cell viability, as shown by a tetrazolium MTT spectrophotometric assay (*p* < 0.001; Figure 1A and Table S1). Addition of noncytotoxic doses of tunicamycin or salubrinal significantly decreased cell viability (*p* < 0.05, combination index (CI) = 0.71 and 0.60, respectively; Figure 1A and Tables S1–S2). As shown by BrdU incorporation, OZ dose-dependently decreased the proliferation rate (*p* < 0.001 for 400 nM OZ; Figure 1B), and the addition of tunicamycin or salubrinal further impeded cell proliferation (*p* < 0.05). OZ induced the activation of executioner caspase-3/7 in HepG2 cells (*p* < 0.001; Figure 1C). Again, addition of tunicamycin or salubrinal further increased caspase-3/7 activity (*p* < 0.001). Although the IRE1 and PERK inhibitors were previously validated [15], these compounds did not affect the sensitivity of HCC cells to 100–400 nM OZ in HepG2 cells. Since tunicamycin increased the sensitivity but is not clinically applicable because of its toxicity, the HIV protease inhibitor nelfinavir, which represents one of the few clinically applicable ER stress-inducing agents [16], was tested. Interestingly, addition of nelfinavir also synergistically increased the sensitivity to OZ (CI = 0.68). MTT viability and BrdU incorporation experiments were repeated in Huh7 cells with similar results (Figure S1A–S1B and Tables S1–S2). These results indicate that the sensitivity of human HCC cells to oprozomib is increased by ER stress inducers.

Next, we questioned whether the efficacy of other proteasome inhibitors, such as the first-in-class proteasome inhibitor bortezomib, could also be enhanced by combination with UPR inducers. A similar increase in antiproliferative efficacy was observed with 25 nM bortezomib in combination with tunicamycin, nelfinavir or salubrinal in HepG2 cells (Figure S2A–S2B). Finally, we assessed whether OZ or bortezomib altered the chemosensitivity of HepG2 cells to 2.5–10.0 μM doxorubicin for 48 h and observed that proteasome inhibition did not alter the chemosensitivity (data not shown). Together, these results indicate that the sensitivity of human HCC cells to proteasome inhibition is enhanced by ER stress signaling. In the next experiments, OZ was applied at a dose of 400 nM, unless otherwise indicated, since this was the concentration at which OZ inhibits the proliferation rate by approximately 50% in both HepG2 and Huh7 cells.

### Antiproliferative effect of oprozomib depends on the build-up of proteotoxic stress

In contrast to the growth-inhibitory effects after prolonged incubation (48 hours, as stated above), 400 nM OZ did not induce a significant alteration in cell viability, proliferation rate or caspase-3/7 activity after shorter incubation times (8–12 hours, data not shown), suggesting that OZ requires sufficient time to build-up proteotoxicity in HCC cells. To demonstrate that proteotoxic stress caused by OZ originates during *de novo* protein synthesis, cycloheximide was used to inhibit protein synthesis. Importantly, treatment with cycloheximide readily increased proliferation (*p* < 0.001; Figure S3A) and cell viability (*p* < 0.01; Figure S3B) and diminished caspase-3/7 activity (*p* < 0.001; Figure S3C) of OZ-challenged HepG2 cells. In addition, we found that OZ increased the levels of ubiquitin-conjugated proteins and that co-incubation with cycloheximide reduced the accumulation of these conjugates (Figure S3D).

Because bortezomib generates oxidative stress, which is crucial for its antitumor activity [17] and is reported to induce ER stress [11], we investigated whether the antiproliferative effect of OZ is also dependent on oxidative stress. Therefore, we measured cell viability and proliferation after treatment with OZ alone or in combination with the antioxidants N-acetyl-L-cysteine (NAC) or ascorbic acid in HepG2 cells. Surprisingly, addition of these antioxidants to OZ did not alter its antitumor action (Figure S3A–S3C). Thus, these findings suggest that the build-up of proteotoxic, and not oxidative, stress is indispensable for the effects of OZ.

### Oprozomib inhibits proteasomal degradation of UPR-mediated proteins without induction of the transcriptional UPR program

**Figure 2:**
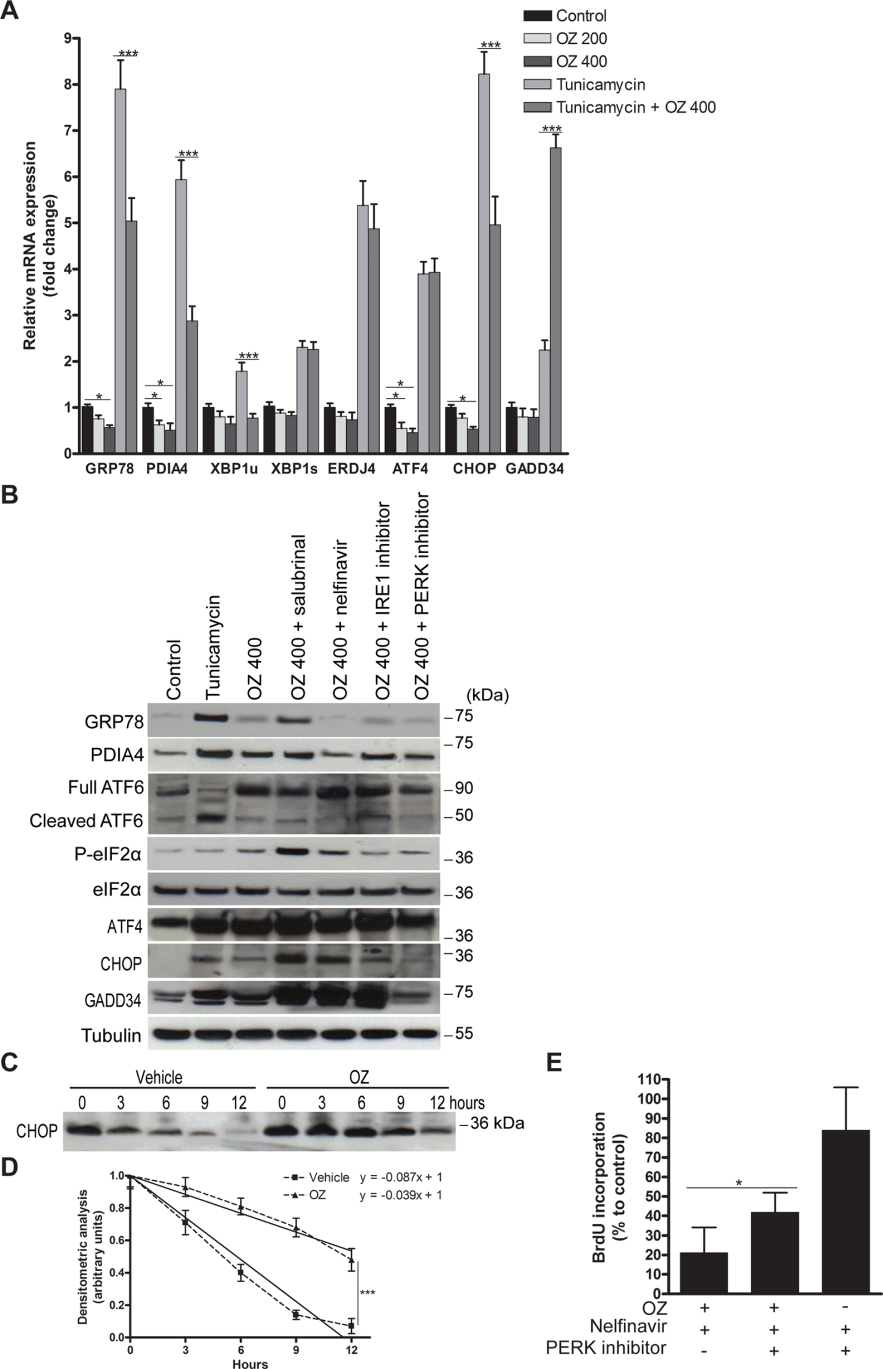
Oprozomib modulates the UPR pattern in HepG2 cells (**A**) Real-time PCR analysis of relative mRNA levels of UPR-mediated genes after 48 hours of incubation with the indicated treatments. (**B**) Immunoblotting of UPR-mediated proteins. (**C**) To measure the half-life of CHOP protein in HepG2 cells, cells were pre-treated for 1 hour with 1 μg/ml tunicamycin and a time-course in the presence of 50 μg/ml cycloheximide, which blocks protein synthesis, was performed. Band intensities were quantified using ImageJ software. (**D**) Half-life of CHOP protein was determined by plotting optical density (arbitrary unit) calculated from densitometric analysis of bands in panel C versus hours of treatment. Data represent the mean ± SD of three independent experiments. (**E**) BrdU incorporation of HepG2 cells incubated with indicated treatments for 48 hours. OZ: 400 nM oprozomib. *p < 0.05, **p < 0.01, ***p < 0.001.

Next, we aimed to evaluate the effects of OZ on the pattern of UPR signaling in HCC cells. First, we examined the induction of UPR targets at the mRNA level by 200 or 400 nM OZ in HepG2 cells. Surprisingly, incubation with 400 nM OZ downregulated the ATF6-mediated *GRP78* and *PDIA4* mRNA levels (*p* < 0.05; Figure 2A) [18]. In addition, incubation with 400 nM OZ repressed *CHOP* and *ATF4* transcription (*p* < 0.05) but did not alter the mRNA levels of IRE1-generated *XBP1s* and its target *ERDJ4*. Even in the presence of ER stress induced by tunicamycin, OZ attenuated the effect of ER stress on the transcription of *GRP78*, *PDIA4*, *XBP1u* and *CHOP*. Intriguingly, the levels of growth arrest and DNA damage inducible 34 (*GADD34*) mRNA, a downstream target of ATF4 and CHOP protein, were increased by incubation with OZ and tunicamycin compared to tunicamycin alone (*p* < 0.001). Collectively, these data indicate that OZ impedes the transcriptional induction of target genes of the ATF6 and PERK pathway without altering the IRE1 RNase activity.

In contrast to the mRNA levels, 400 nM OZ increased the protein levels of GRP78, PDIA4, ATF4 and CHOP (Figure 2B). In line with its transcriptional activation, GADD34 protein levels were also elevated. Intrigued by these findings, we determined the protein half-life of the transcription factor CHOP in HepG2 cells by evaluating its time-course in the presence of cycloheximide (Figure 2C). The half-life of CHOP protein increased from 5.75 hours in vehicle-treated to 12.82 hours in OZ-treated cells (*p* < 0.001; Figure 2D). Thus, OZ increased the UPR-regulated CHOP protein levels by inhibition of its proteasomal degradation and not by enhanced *de novo* synthesis following induction of the transcriptional UPR program.

### OZ impedes tunicamycin-induced cytoprotective ATF6 signaling through direct RIP inhibition

Because OZ increased the levels of full ATF6 protein without changing the levels of the transcriptionally active ATF6 fragment (Figure 2B), the modulatory effect of OZ on tunicamycin-mediated activation of ATF6 was explored (Figure S4). First, we assessed the effect of OZ on the tunicamycin-mediated transcriptional induction of additional ATF6-regulated UPR targets, such as *GRP94*, *ERO1L*, *CALR* and *HERPUD1* mRNA (Figure S4A) [12, 18]. As expected, upregulation of all ATF6 targets by tunicamycin was evident. Addition of OZ to tunicamycin downregulated these selected ATF6 targets. To assess the effect of OZ on the RIP, the processing of another target of RIP, sterol regulatory element binding protein-1 (SREBP-1), was examined. Western blotting confirmed the accumulation of precursor SREBP-1 in OZ-treated HepG2 cells (Figure S4B). These results suggest that OZ inhibits ATF6 signaling by RIP inhibition.

RIP inhibition could occur either directly by inhibition of the S1P or S2P expression or activity or indirectly by upregulation of a repressor of protease-mediated ATF6 activation. OZ did not alter the *S1P* or *S2P* mRNA levels (Figure S4C), suggesting that OZ functions through post-translational RIP inhibition without affecting *S1P* or *S2P* expression. Nucleobindin 1 is a reported ATF6 repressor [19]. While tunicamycin increased nucleobindin 1 expression, the expression in OZ-treated cells was indistinguishable from vehicle-treated cells (Figure S4B).

Treatment of HepG2 cells with 25 μM 1,10-phenanthroline, a metalloprotease-specific S2P inhibitor [20], led to accumulation of precursor SREBP-1 and to the absence of processed SREBP-1 detection and did not alter the nucleobindin 1 expression (Figure S4B). In addition, 5 to 50 μM of 1,10-phenanthroline dose-dependently reduced the HepG2 cell viability (Figure S4D), phenocopying the effects of OZ (Figure 1 and S4B, respectively). Finally, also 1,10-phenanthroline at 25 μM did not significantly alter the S2P transcription (Figure S4E), suggesting that inhibition of the activity of S2P does not affect its expression. Thus, OZ, at an effective dose of 400 nM, inhibits tunicamycin-induced cytoprotective ATF6 signaling by RIP inhibition likely via direct off-target inhibition of S1/S2 protease activity without altering their expression.

### The PERK pathway regulates the nelfinavir-mediated increase in sensitivity to OZ

OZ slightly increased the eIF2α phosphorylation, which could not be inhibited by the PERK inhibitor (Figure 2B), pointing to the involvement of other eIF2α kinases, as previously reported for eIF2α phosphorylation induced by proteasome inhibitor MG-132 [21]. Importantly, addition of salubrinal or nelfinavir profoundly increased the OZ-induced eIF2α phosphorylation and pro-apoptotic CHOP protein levels (Figure 2B), which may contribute to the increased sensitivity to OZ (Figure 1). Indeed, addition of the PERK inhibitor increased the proliferation rate of HepG2 cells treated with the combination of OZ and nelfinavir (*p* < 0.05; Figure 2E), validating the role of the PERK pathway in the mechanism of this combination. Addition of the IRE1 inhibitor did not alter the proliferation rate (data not shown). Furthermore, addition of nelfinavir to OZ abolished the protein levels of the ATF6-dependent chaperones GRP78 and PDIA4 (Figure 2B), possibly exacerbating the generated proteotoxicity. Interestingly, nelfinavir was previously reported to induce apoptosis in liposarcoma cells by direct S2P inhibition [22]. These data suggest that the PERK pathway activation is a key mechanism inducing the enhanced anti-tumor activity of OZ following the addition of UPR activators.

### OZ reduced tumor burden in orthotopic and xenograft mouse models for HCC

Prior to evaluating the antitumor activity of OZ in the DEN-induced mouse model characterized by severe liver dysfunction [23], different 2-week dosing regimens were tested in 25-week saline-treated and DEN-treated mice (*n* = 3, Table 1). We observed high mortality in the mice with liver dysfunction treated with 50 mg/kg/day for 5 consecutive days per week. However, at 30 mg/kg/day for 3 consecutive days per week, no mortality occurred. Therefore, this dosing regimen was applied in the following experiments.

**Table 1:** Mortality at different dosing regimens (*n* = 3 in each group)

Consecutive-day dosing in weekly cycle	Mortality in saline-treated mice	Mortality in DEN-treated mice
50 mg/kg/day for 5 days	2/3	3/3
30 mg/kg/day for 5 days	1/3	1/3
30 mg/kg/day for 4 days	0/3	1/3
30 mg/kg/day for 3 days	0/3	0/3

OZ for 4 weeks did not affect mortality in 25-week saline- or DEN-injected mice (*n* = 12, Table 2). The average body weight of mice was lower following 25 weeks of DEN compared to saline administration (*p* < 0.001, Table 2). Subsequent treatment with OZ did not alter the average body weight compared to vehicle. Although serum ALT and AST levels were elevated by DEN administration (*p* < 0.001), treatment with OZ did not alter these levels in the surviving mice (Figure S5A).

**Table 2:** Average body weight (g) ± SD and survival of mice (*n* = 12 in each group)

Group	Average body weight 25 weeks (g)	Average body weight 29 weeks (g)	Survival (%)
Saline => vehicle	27.22 ± 1.32	26.03 ± 1.61	100
DEN => vehicle	19.75 ± 1.76^***^	18.30 ± 2.92	58.33
DEN => OZ	20.86 ± 1.45	19.17 ± 2.10 NS	58.33
DEN => OZ + nelfinavir	22.96 ± 3.65	17.92 ± 3.19 NS	66.67
DEN => OZ + salubrinal	21.23 ± 5.89	18.09 ± 6.23 NS	66.67

*** *p* < 0.001: 25 weeks DEN vs. saline, NS = not significant compared to vehicle.

**Figure 3:**
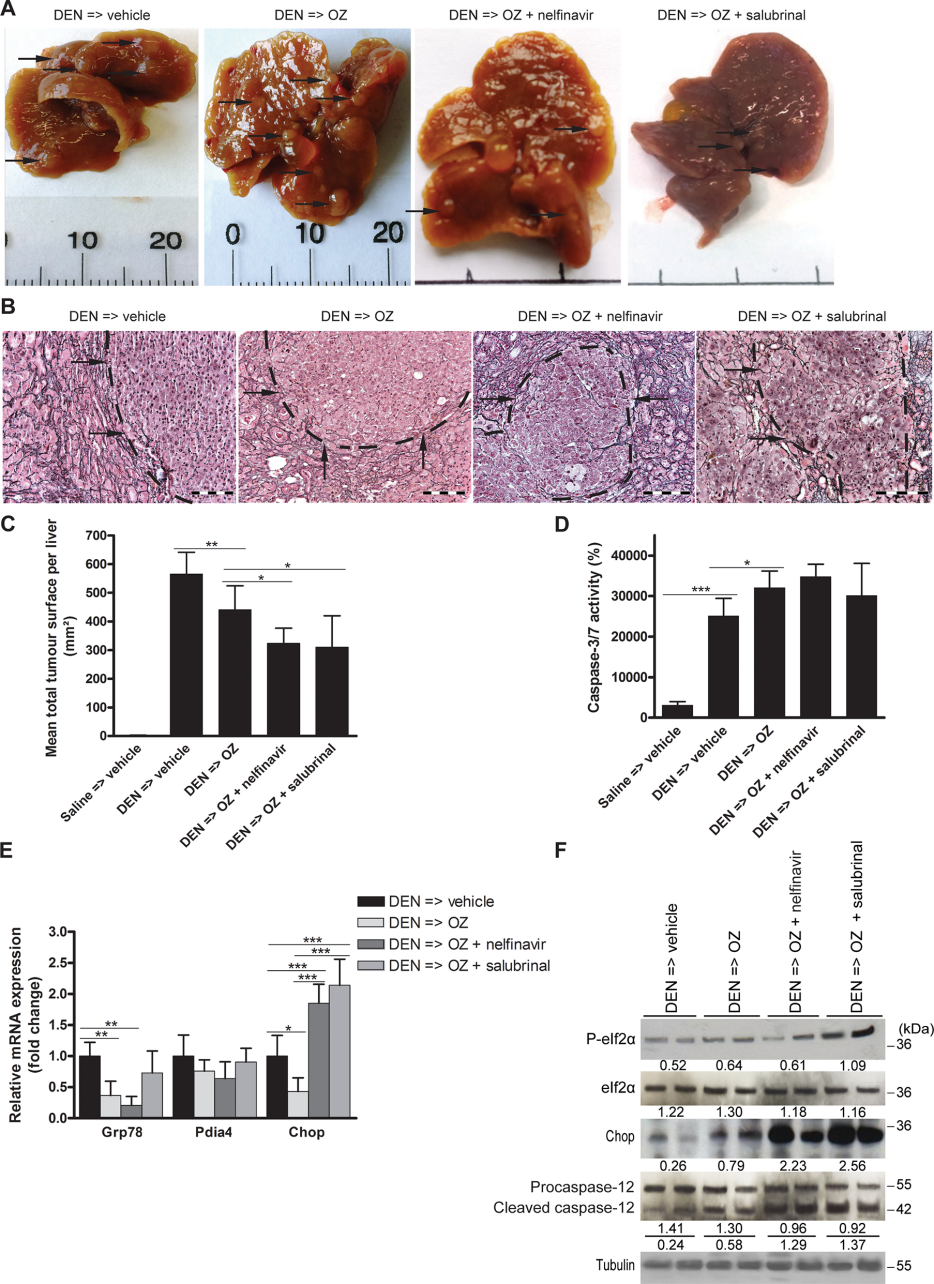
Impact of oprozomib in monotherapy or in combination with ER stress modulators on an orthotopic and a xenograft model for HCC (**A**) Photographs of representative livers after different treatments. (**B**) Reticulin staining. Scale bar: 100 μm. (**C**) Assessment of tumor burden in the carcinogen-induced mouse model in randomly selected high-power fields. (**D**) Hepatic caspase-3/7 activity *ex vivo*. Values represent the mean ± SD. (**E**) Real-time PCR analysis and (**F**) Immunoblotting of UPR targets in isolated DEN-induced tumors following the indicated treatments. Densitometric analysis relative to tubulin is indicated below. *p < 0.05, **p < 0.01, ***p < 0.001.

DEN-treated mice that received OZ developed fewer macroscopic nodules per liver (all sizes: 16.2 ± 4.5 after vehicle versus 11.1 ± 3.9 after OZ; *p* < 0.05). HCC burden, microscopically quantified by the loss of reticulin staining, was reduced in OZ-treated compared to vehicle-treated mice (*p* < 0.01, Figure 3A–3C). Hepatic caspase-3/7 activity levels *ex vivo*, which were elevated by DEN compared to saline administration (*p* < 0.001, Figure 3D), were increased following OZ monotherapy compared to vehicle-treated HCC-bearing mice (*p* < 0.05), consistent with *in vivo* apoptosis induction.

Since we previously reported the UPR pattern in the DEN-induced mouse model [15], we examined the impact of OZ administration on the expression of UPR markers in HCC. OZ administration reduced the levels of *Grp78* (*p* < 0.01) and *Chop* (*p* < 0.05) mRNA in the isolated tumors (Figure 3E). However, immunoblotting of lysates of isolated tumors revealed that OZ was associated with higher CHOP protein levels *in vivo* (Figure 3F). In contrast, UPR-regulated caspase-12 cleavage was only slightly increased by OZ. These observations provide evidence that the efficacy of OZ in inhibiting the growth of DEN-induced HCC is through similar mechanisms as those observed *in vitro*.

**Figure 4:**
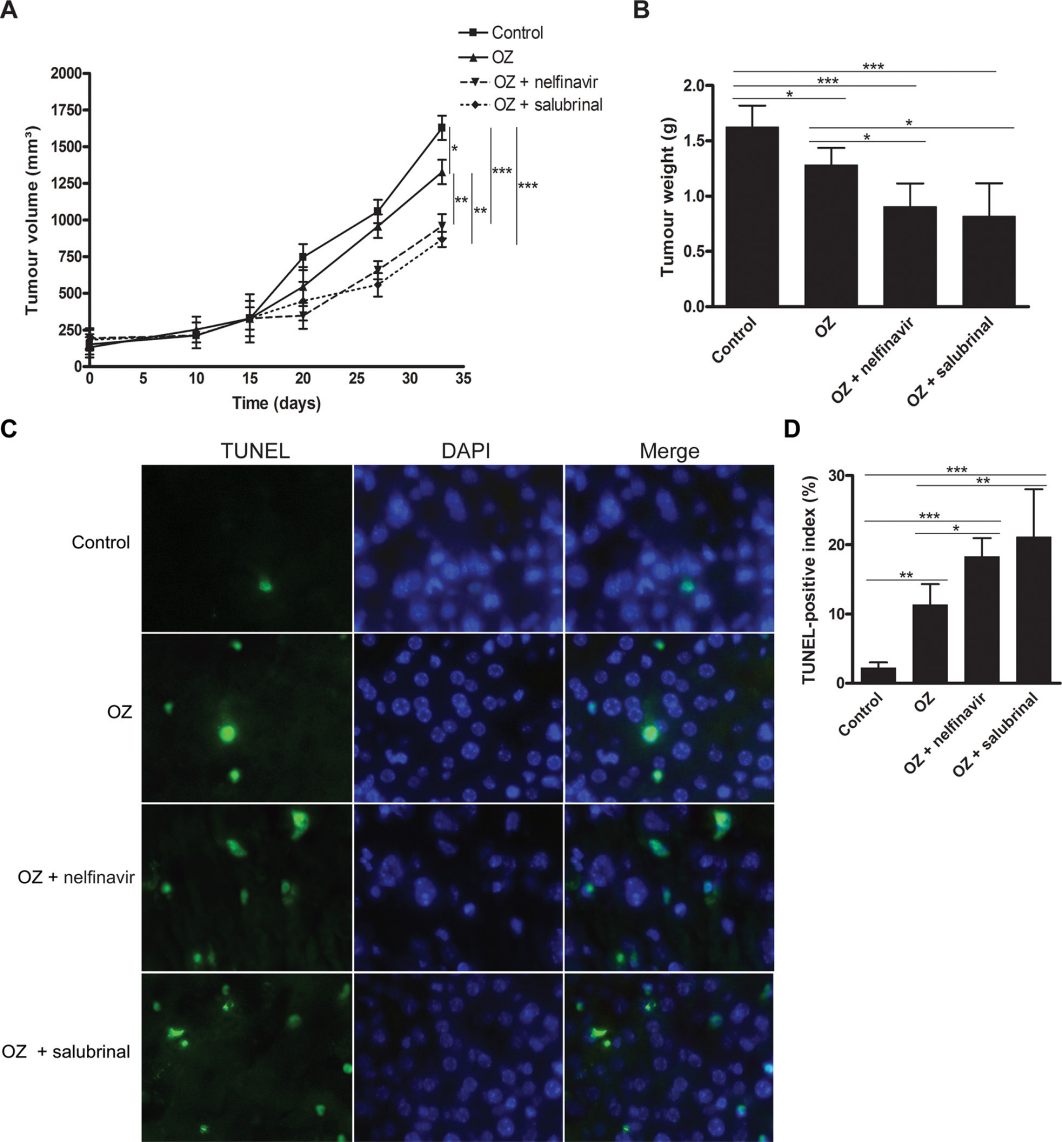
Oprozomib and UPR modulation in experimental HCC (**A**) Evolution of tumor volume in mice bearing HepG2-derived xenograft tumors. (**B**) Final tumor weights. (**C**) TUNEL immunofluorescence in HepG2 xenografts and (**D**) quantification of TUNEL-positive index (*n* = 6). OZ: oprozomib. Values represent the mean ± SD. *p < 0.05, **p < 0.01, ***p < 0.001.

Secondly, the effect of OZ was assessed in a HepG2 xenograft model. No significant differences in body weight or appearance between control and OZ-treated animals were observed during the course of the xenograft study (data not shown). OZ administration suppressed the growth rate of the HepG2-derived tumors (*p* < 0.05, Figure 4A). Accordingly, TUNEL immunofluorescence demonstrated a significant increase of TUNEL-positive apoptotic HepG2 cells following OZ administration (*p* < 0.01, Figure 4C–4D).

### Nelfinavir and salubrinal potentiate the therapeutic efficacy of OZ in experimental HCC

Next, we assessed the effects of supplementary UPR activation via nelfinavir and salubrinal on HCC growth *in vivo*. Administration of nelfinavir or salubrinal in mice after 25 weeks of DEN induction did not alter the mean body weight compared to vehicle (Table 1). Although hepatic caspase-3/7 activity was not significantly increased (Figure 3D), combining OZ with nelfinavir was more efficacious compared to OZ monotherapy in the orthotopic model (number of macroscopic nodules per liver: 11.1 ± 3.9 after OZ versus 7.2 ± 5.2 after OZ and nelfinavir; microscopic tumor burden: *p* < 0.05; Figure 3A–3C). Addition of salubrinal at 1 mg/kg/24 hours similarly improved the effect of OZ (number of macroscopic nodules per liver: 6.7 ± 4.8 after OZ and salubrinal; microscopic tumor burden: *p* < 0.05; Figure 3A–3C). Interestingly, addition of nelfinavir or salubrinal to OZ promoted eIf2α phosphorylation and downstream *Chop* mRNA and protein levels (Figure 3E–3F), suggesting intensified pro-apoptotic UPR signaling. Caspase-12 cleavage was indeed markedly increased by the addition of nelfinavir or salubrinal to OZ treatment (Figure 3F), confirming the pronounced induction of UPR-triggered apoptosis. In contrast, salubrinal at 1 mg/kg/72 hours induced no detectable effects on the antitumor efficacy of OZ, eIf2α phosphorylation or Chop expression (data not shown).

**Figure 5:**
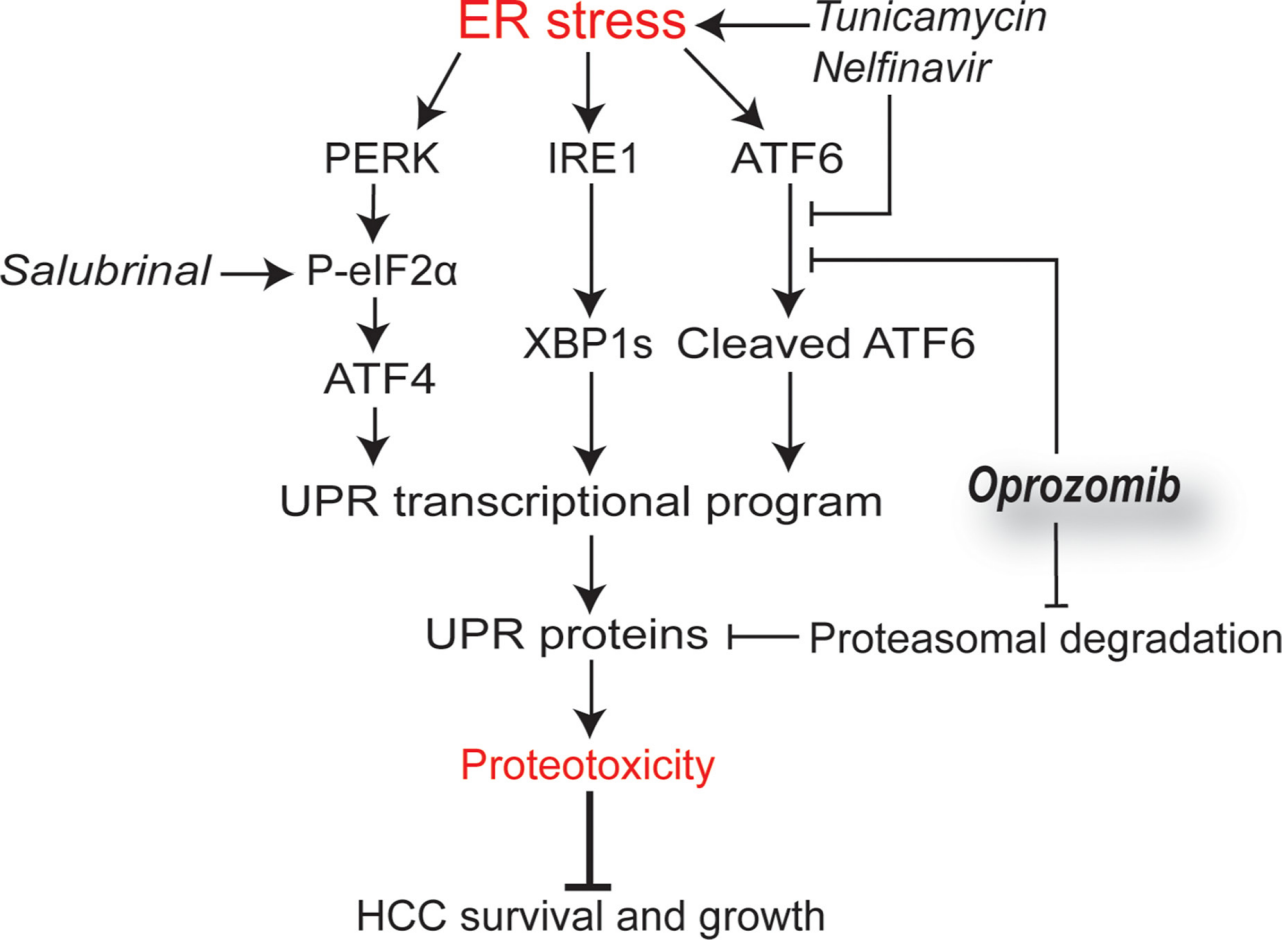
Schematic model outlining the mechanisms of oprozomib with indication of the point of action of the applied products Persistent ER stress activates the tripartite UPR-mediated transcriptional program followed by translation of these UPR proteins, which leads to proteotoxicity-mediated tumor cell death. Although oprozomib did not induce the UPR and even inhibited ATF6-mediated transcription, it increased the UPR-mediated protein levels by prolonging their half-life. This UPR dysregulation allows for enhanced proteotoxicity through supplementary boosting PERK activity by tunicamycin, nelfinavir (also inhibits ATF6) or salubrinal.

Accordingly, in the HepG2 xenograft model, dual therapy with OZ and nelfinavir or OZ and salubrinal effectively inhibited tumor growth compared to vehicle-treated (both *p* < 0.001) and OZ-treated mice (both *p* < 0.01) (Figure 4A), whereas nelfinavir or salubrinal monotherapy did not alter the xenograft growth (Figure S5B–S5C). Furthermore, addition of nelfinavir or salubrinal augmented the number of TUNEL-positive HepG2 cells in the xenograft tumors compared to OZ monotherapy (*p* < 0.05 and *p* < 0.01, respectively, Figure 4C–4D), suggesting robust induction of apoptosis when UPR modulators are added. Thus, we identified that nelfinavir and salubrinal potentiate the therapeutic efficacy of OZ in different models for HCC, likely through increased UPR-mediated apoptosis via induction of Chop synthesis while OZ diminishes its proteasomal degradation (Figure 5).

## DISCUSSION

Proteasome inhibition could represent a novel therapeutic strategy for advanced HCC [5, 7]. The next-generation orally bio-available irreversible proteasome inhibitor oprozomib (OZ) is assumed to evoke fewer adverse events and improve antitumor activity compared to the first-in-class proteasome inhibitor bortezomib [3]. Recently, OZ was shown to reduce tumor growth in myeloma and head and neck cancer xenograft models [3]. In this study, OZ exerted potent antitumor effects *in vitro* and in different *in vivo* models for HCC, supporting the potential value of irreversibly targeting the proteasome in the treatment of HCC. Moreover, we revealed a strategy to enhance the efficacy of OZ through modulation of the UPR.

When unfolded proteins accumulate in the ER, the UPR is initiated to allow the cells to restore homeostasis through proteasomal degradation of unfolded proteins, translational arrest and promoting protein folding capacity [11]. The association between UPR activity and therapeutic efficacy of proteasome inhibition was first illustrated in myeloma, in which patient serum levels of XBP1 correlated with the clinical response towards bortezomib [24]. Theoretically, decreased protein degradation by proteasome inhibition could lead to oxidative stress and UPR activation. However, the effect on the UPR is not well understood. We showed that the cytotoxic effect of OZ depends on the build-up of proteotoxic stress without an important contribution of oxidative stress. Although OZ did not induce the transcriptional UPR program and even inhibited the protease-dependent activation of the cytoprotective ATF6 pathway, OZ increased the protein levels of different UPR markers. Notably, OZ increased the protein stability of the pro-apoptotic transcription factor CHOP and induced transcription of *GADD34*. Thus, OZ increased the levels of the UPR-regulated proteins by decreasing their proteasomal degradation rather than generally activating the UPR program due to increased unfolded protein load. Apparently, rapid proteasomal degradation of UPR effector proteins is a negative feedback mechanism following recovery of ER proteostasis.

Consistent with the effect of OZ on the UPR, PERK or IRE1 inhibition did not alter its effect, whereas ER stress inducers, such as tunicamycin or nelfinavir, or an inhibitor of eIF2α dephosphorylation enhanced the growth-inhibitory effects of OZ in HCC cells. Interestingly, a similar synergy was observed with bortezomib, suggesting this concept can also be applied to other proteasome inhibitors. Because the maximum serum levels attained in patients are much higher (bortezomib: 0.16 μM (1.3 mg/m^2^ intravenous); oprozomib: 3.8 μM (30 mg per os) [25]), the concentrations used *in vitro* are clinically relevant.

Based on these results, we evaluated the *in vivo* effects of the combination of nelfinavir or salubrinal and OZ in HCC. Both nelfinavir and salubrinal enhanced the growth-inhibitory effect without cumulative toxicity, suggesting this rational combination presents a safe strategy to potentiate the antitumor effects of proteasome inhibition in HCC. Experience in the use of proteasome inhibitors and nelfinavir in clinical practice and the oral bio-availability are considerable advantages for implementation.

Bortezomib resistance in myeloma cells was previously linked to eIF2α phosphorylation [26]. Salubrinal typically induces enhanced resistance to stress conditions, such as those triggered by oxidizing or UPR-activating agents [27, 28]. However, here we provide evidence that salubrinal renders HCC cells more susceptible to OZ-mediated proteasome inhibition by stimulating the PERK/phospho-eIF2α/CHOP pathway. Of note, human HCC, in contrast to unaffected adjacent liver tissue, is characterized by increased CHOP staining [29], suggesting that tumor-selective effects can be achieved. Additionally, we previously reported the high expression of Chop in the HCC nodules compared to the non-HCC tissue of the DEN-induced mouse model [15].

Although a recent phase I trial with OZ demonstrated that OZ has an acceptable safety profile when given daily for 5 consecutive days every 2 weeks in patients with solid tumors [30], significant toxicity occurred when OZ was administered for several consecutive days in mice with DEN-induced liver dysfunction. However, identification of sensitivity enhancers by UPR modulation could allow for a reduction of dose and dose-related toxicity. Because ER stress potentiates the antitumor efficacy of OZ, we speculate that OZ exerts a stronger effect on hypoxic ER-stressed tumor cells compared to the normal liver tissue [15]. Consequently, OZ could be more efficacious in combination with antiangiogenic treatments promoting tumor hypoxia-induced UPR [11, 31].

In conclusion, dysregulation of the transcriptional UPR program and reduced proteasomal degradation of pro-apoptotic UPR-mediated proteins are involved in OZ-induced cell death. Moreover, modulation of the interplay between OZ and the UPR enhances its antitumor efficacy without cumulative toxicity. Therefore, OZ monotherapy or OZ in combination with an UPR modulator may present a novel and clinically applicable therapeutic strategy for HCC.

## MATERIALS AND METHODS

### Cell culture

HepG2 (ATCC, Virginia, USA) and Huh7 cells (JCRB, Japan) were cultured in Dulbecco's Modified Eagle's medium supplemented with 10% fetal bovine serum (Life Technologies, Ghent, Belgium). Cells were incubated for 48 hours with oprozomib (100–400 nM; ApexBio, USA), tunicamycin (1 μg/mL; Sigma, Diegem, Belgium), PERK inhibitor (14 μM; GSK2656157, Chengdu novi, Shandong, China), salubrinal (50 μM; Sigma), IRE1 inhibitor (25 μM; 4 μ8C, Calbiochem, Massachusetts, USA), cycloheximide (5 μM; Sigma), 1,10-phenanthroline (5–50 μM; Sigma), ascorbic acid (50 μM; Sigma), nelfinavir (10 μM; Sigma), doxorubicin hydrochloride (2.5–10.0 μM; Sigma) or pre-treated with N-acetyl-L-cysteine (2 hours; 5 μM; Sigma) and compared to equal volumes of solvent as control. Each condition was performed in quadruplicate.

### Animals

Wild-type 129S2/SvPasCrl mice were purchased from Charles River, Belgium, and were housed as previously described [13]. The animals had free access to water and to a commercial chow (standard maintenance chow, Pavan Service-Carfil, Oud-Turnhout, Belgium). Five-week-old male mice received weekly intraperitoneal injections with saline or diethylnitrosamine (DEN) (35 mg/kg, Sigma) for 25 weeks. Then, 4 DEN-treated groups (*n* = 12 for each group) were treated for 4 weeks with oprozomib (intragastric 30 mg/kg/day for 3 consecutive days per week) alone or in combination with salubrinal (intraperitoneally 1 mg/kg/day) or nelfinavir (intraperitoneally 250 mg/kg/day) and compared to a similar volume of vehicle. Blood was collected from the retro-orbital sinus under isoflurane anesthesia. After macroscopic evaluation and quantification of the number of hepatic tumors, all organs were fixed in 4% phosphate-buffered formaldehyde (Klinipath, Olen, Belgium) and embedded in paraffin or snap frozen in liquid nitrogen. Hematoxylin/eosin and reticulin staining were performed to assess tumor burden, and the results were blindly evaluated by two independent observers (CC and YV).

For the xenograft model, HepG2 cells (6 × 10^6^) were suspended in 100 μl serum-free media and mixed with 100 μl Matrigel (BD Biosciences, Bedford, MA, USA). Cell/Matrigel mixture was injected subcutaneously into the right flank of 8-week-old athymic nude (Foxn1nu/Foxn1nu) mice housed in filter-topped cages. Tumor dimensions were recorded three times per week with a digital caliper starting with the first day of treatment. Tumor volumes were calculated using the following formula: volume (mm³) = ab²/2, where b was the smaller dimension. When tumors reached 150 mm³, animals were randomized into four groups (*n* = 6) to receive the same treatment regimens as the DEN-treated mice plus monotherapy with salubrinal (intraperitoneally 1 mg/kg/day) or nelfinavir (intraperitoneally 250 mg/kg/day). The ethical committee of experimental animals at Ghent University approved the protocols (ECD 13/39).

Detailed information of MTT, TUNEL, caspase-3/7 activity, bromodeoxyuridine (BrdU) incorporation assays, total RNA extraction, quantitative real-time PCR and Western blotting is provided in the Supplementary Materials and Methods.

### Statistics

Statistical analyses were performed using SPSS 21 (SPSS, Chicago, USA). Data are presented as the mean ± SD or percentage relative to controls. Variables were tested for normality using the Shapiro-Wilk test. Normally distributed data were subjected to the unpaired Student's *t*-test. Data involving more than two groups were assessed by one-way analysis of variance (ANOVA) with Bonferroni's post-hoc test. Non-normally distributed data were tested using the Mann-Whitney *U* test. The chi-squared test was used to compare mortality. The IC50 values were obtained using the Bliss method. Interpretation of CI values, as calculated by the method of Chou and Talalay [14] using CompuSyn software (ComboSyn Inc., Paramus, NJ, USA), is defined such that CI = 1 indicates an additive effect, and CI < 1 and CI > 1 indicate synergism and antagonism, respectively. Reported *p*-values were two-sided and considered significant when less than 0.05.

## SUPPLEMENTARY MATERIALS FIGURES AND TABLES



## Abbreviations

HCC: hepatocellular carcinoma
ER: endoplasmic reticulum
GRP78: glucose-regulated protein, 78 kDa
OZ: oprozomib
UPR: unfolded protein response
PERK: PKR-like endoplasmic reticulum kinase
IRE1: inositol-requiring enzyme-1
ATF6: activating transcription factor 6
eIF2α: eukaryotic initiation factor 2α
ATF4: activating transcription factor 4
CHOP: c/EBP-homologous protein
XBP1u: unspliced X-box-binding protein 1
XBP1s: spliced X-box-binding protein 1
ERDJ4: endoplasmic reticulum DnaJ homolog 4
RIP: intramembrane proteolysis
S1P: site-1 protease
S2P: site-2 protease
GRP94: glucose-regulated protein, 94 kDa
PDIA4: protein disulfide isomerase A4
CALR: calreticulin
ERO1L: endoplasmic oxidoreductin-1-like protein
HERPUD1: homocysteine-inducible, ER stress-inducible, ubiquitin-like domain member 1
DEN: diethylnitrosamine
NAC: N-acetyl-L-cysteine
GADD34: growth arrest and DNA damage inducible 34
SREBP-1: sterol regulatory element binding protein-1
CI: combination index

## References

[1] Ferlay J, Soerjomataram I, Ervik M, Dikshit R, Eser S, Mathers C, Rebelo M, Parkin DM, Forman D, Bray F (2013). GLOBOCAN 2012 v1.0, Cancer Incidence and Mortality Worldwide: IARC CancerBase [Internet]. http://globocan.iarc.fr/.

[2] EASL-EORTC clinical practice guidelines: management of hepatocellular carcinoma (2012). J. Hepatol.

[3] Mateos MV, Ocio EM, San Miguel JF (2013). Novel generation of agents with proven clinical activity in multiple myeloma. Semin. Oncol.

[4] Aronson LI, Davies FE (2012). DangER: protein ovERload. Targeting protein degradation to treat myeloma. Haematologica.

[5] Saeki I, Terai S, Fujisawa K, Takami T, Yamamoto N, Matsumoto T, Hirose Y, Murata Y, Yamasaki T, Sakaida I (2012). Bortezomib induces tumor-specific cell death and growth inhibition in hepatocellular carcinoma and improves liver fibrosis. J. Gastroenterol.

[6] Boozari B, Mundt B, Woller N, Strüver N, Gürlevik E, Schache P, Kloos A, Knocke S, Manns MP, Wirth TC, Kubicka S, Kühnel F (2010). Antitumoural immunity by virus-mediated immunogenic apoptosis inhibits metastatic growth of hepatocellular carcinoma. Gut.

[7] Kim GP, Mahoney MR, Szydlo D, Mok TSK, Marshke R, Holen K, Picus J, Boyer M, Pitot HC, Rubin J, Philip PA, Nowak A, Wright JJ (2012). An international, multicenter phase II trial of bortezomib in patients with hepatocellular carcinoma. Invest. New Drugs.

[8] Saini N, Mahindra A (2013). Therapeutic strategies for the treatment of multiple myeloma. Discov. Med.

[9] Zhou HJ, Aujay MA, Bennett MK, Dajee M, Demo SD, Fang Y, Ho MN, Jiang J, Kirk CJ, Laidig GJ, Lewis ER, Lu Y, Muchamuel T (2009). Design and synthesis of an orally bioavailable and selective peptide epoxyketone proteasome inhibitor (PR-047). J. Med. Chem.

[10] Stewart AK, Rajkumar SV, Dimopoulos MA, Masszi T, Špička I, Oriol A, Hájek R, Rosiñol L, Siegel DS, Mihaylov GG, Goranova-Marinova V, Rajnics P, Suvorov A (2014). Carfilzomib, Lenalidomide, and Dexamethasone for Relapsed Multiple Myeloma. N. Engl. J. Med.

[11] Vandewynckel Y-P, Laukens D, Geerts A, Bogaerts E, Paridaens A, Verhelst X, Janssens S, Heindryckx F, Van Vlierberghe H (2013). The paradox of the unfolded protein response in cancer. Anticancer Res.

[12] Rachidi S, Sun S, Wu BX, Jones E, Drake RR, Ogretmen B, Cowart A, Clarke CJ, Hannun YA, Chiosis G, Liu B, Li Z (2014). Endoplasmic reticulum heat shock protein gp96 maintains liver homeostasis and promotes hepatocellular carcinogenesis. J. Hepatol.

[13] Van de Veire S, Stalmans I, Heindryckx F, Oura H, Tijeras-Raballand A, Schmidt T, Loges S, Albrecht I, Jonckx B, Vinckier S, Van Steenkiste C, Tugues S, Rolny C (2010). Further pharmacological and genetic evidence for the efficacy of PlGF inhibition in cancer and eye disease. Cell.

[14] Chou TC (2010). Drug combination studies and their synergy quantification using the Chou-Talalay method. Cancer Res.

[15] Vandewynckel Y-P, Laukens D, Bogaerts E, Paridaens A, Van den Bussche A, Verhelst X, Van Steenkiste C, Descamps B, Vanhove C, Libbrecht L, De Rycke R, Lambrecht BN, Geerts A (2015). Modulation of the unfolded protein response impedes tumor cell adaptation to proteotoxic stress: a PERK for hepatocellular carcinoma therapy. Hepatol. Int.

[16] Brüning A (2011). Analysis of nelfinavir-induced endoplasmic reticulum stress. Methods Enzymol.

[17] Ling YH, Liebes L, Zou Y, Perez-Soler R (2003). Reactive oxygen species generation and mitochondrial dysfunction in the apoptotic response to Bortezomib, a novel proteasome inhibitor, in human H460 non-small cell lung cancer cells. J. Biol. Chem.

[18] Shoulders MD, Ryno LM, Genereux JC, Moresco JJ, Tu PG, Wu C, Yates JR, Su AI, Kelly JW, Wiseman RL (2013). Stress-Independent Activation of XBP1s and/or ATF6 Reveals Three Functionally Diverse ER Proteostasis Environments. Cell Rep.

[19] Tsukumo Y, Tomida A, Kitahara O, Nakamura Y, Asada S, Mori K, Tsuruo T (2007). Nucleobindin 1 controls the unfolded protein response by inhibiting ATF6 activation. J. Biol. Chem.

[20] Feng L, Yan H, Wu Z, Yan N, Wang Z, Jeffrey PD, Shi Y (2007). Structure of a site-2 protease family intramembrane metalloprotease. Science.

[21] Yerlikaya A, Kimball SR, Stanley BA (2008). Phosphorylation of eIF2alpha in response to 26S proteasome inhibition is mediated by the haem-regulated inhibitor (HRI) kinase. Biochem. J.

[22] Guan M, Fousek K, Jiang C, Guo S, Synold T, Xi B, Shih CC, Chow WA (2011). Nelfinavir induces liposarcoma apoptosis through inhibition of regulated intramembrane proteolysis of SREBP-1 and ATF6. Clin. Cancer Res.

[23] Heindryckx F, Mertens K, Charette N, Vandeghinste B, Casteleyn C, Van Steenkiste C, Slaets D, Libbrecht L, Staelens S, Starkel P, Geerts A, Colle I, Van Vlierberghe H (2010). Kinetics of angiogenic changes in a new mouse model for hepatocellular carcinoma. Mol. Cancer.

[24] Ling SCW, Lau EKK, Al-Shabeeb A, Nikolic A, Catalano A, Iland H, Horvath N, Ho PJ, Harrison S, Fleming S, Joshua DE, Allen JD (2012). Response of myeloma to the proteasome inhibitor bortezomib is correlated with the unfolded protein response regulator XBP-1. Haematologica.

[25] Papadopoulos KP, Mendelson DS, Tolcher AW, Patnaik A, Burris HA, Rasco DW, Bendell JC, Gordon MS, Kato G, Wong H, Bomba D, Lee S, Gillenwater HH (2011). A phase I, open-label, dose-escalation study of the novel oral proteasome inhibitor (PI) ONX 0912 in patients with advanced refractory or recurrent solid tumors. J. Clin. Oncol.

[26] Schewe DM, Aguirre-Ghiso JA (2009). Inhibition of eIF2alpha dephosphorylation maximizes bortezomib efficiency and eliminates quiescent multiple myeloma cells surviving proteasome inhibitor therapy. Cancer Res.

[27] Boyce M, Bryant KF, Jousse C, Long K, Harding HP, Scheuner D, Kaufman RJ, Ma D, Coen DM, Ron D, Yuan J (2005). A selective inhibitor of eIF2alpha dephosphorylation protects cells from ER stress. Science.

[28] Lewerenz J, Maher P (2009). Basal levels of eIF2alpha phosphorylation determine cellular antioxidant status by regulating ATF4 and xCT expression. J. Biol. Chem.

[29] Dezwaan-McCabe D, Riordan JD, Arensdorf AM, Icardi MS, Dupuy AJ, Rutkowski DT (2013). The Stress-Regulated Transcription Factor CHOP Promotes Hepatic Inflammatory Gene Expression, Fibrosis, and Oncogenesis. PLoS Genet.

[30] Wang H, Guan F, Chen D, Dou QP, Yang H (2014). An analysis of the safety profile of proteasome inhibitors for treating various cancers. Expert Opin. Drug Saf.

[31] Paridaens A, Laukens D, Vandewynckel Y-P, Coulon S, Van Vlierberghe H, Geerts A, Colle I (2014). Endoplasmic reticulum stress and angiogenesis: is there an interaction between them?. Liver Int.

