# Supplementary material for: Next-generation proteasome inhibitor oprozomib synergizes with modulators of the unfolded protein response to suppress hepatocellular carcinoma
Source: Oncotarget. 2016 May 7;7(23):34988–5000. doi: 10.18632/oncotarget.9222 (PMC5085204; doi:10.18632/oncotarget.9222)
Supplement: Supplementary file 1 [file oncotarget-07-34988-s001.pdf]

Next-generation proteasome inhibitor oprozomib synergizes with modulators of the unfolded protein response to suppress hepatocellular carcinoma

Supplementary Materials

SUPPLEMENTARY MATERIAL AND METHODS

MTT viability assays

Cells were seeded in 96-well plates, at 5×10^4 cells/well in Dulbecco's Modified Eagle's medium supplemented with 10% fetal bovine serum and incubated the next day with the indicated products. After 48 hours, cell viability was determined using the MTT assay (Roche Diagnostics, Mannheim, Germany). After supernatant collection, the reagent was added, and the cells were incubated for 4 hours at 37°C. The absorbance of the DMSO-solved MTT crystals was measured at 450 nm against a background control at 655 nm (Multiskan Ascent, Leuven, Belgium). Experiments were performed in quadruplicate.

TUNEL apoptosis assay

Embedded liver sections were deparaffinized, rehydrated through graded alcohol, and permeabilized with 0.1% TritonX-100 at room temperature (8 min incubation). Slides were rinsed twice in a phosphate-buffered saline (PBS). Apoptosis was detected through in situ terminal deoxynucleotidyl transferase (TdT)-mediated deoxyuridine triphosphate (dUTP) nick end-labeling (TUNEL), using the In Situ Cell Death Detection Kit (Roche, Vilvoorde, Belgium). Slides were incubated with the TUNEL reaction mixture containing TdT and fluorescein-dUTP for 1 h at 37°C; and subsequently rinsed three times in PBS. The sections were mounted with an antifade solution containing 4′,6-diamidino-2-phenylindole (DAPI) (Vectashield, Lab Consult) for nuclear staining. Images were acquired on a Nikon TE300 inverted epifluorescence microscope with a x20 objective and equipped with a Nikon DS-Ri1 cooled color CCD camera (Nikon Belux, Brussel, Belgium). For quantification of apoptotic cells, five random areas from each slide and 5 slides per liver were analyzed. Cells containing green fluorescence and either nuclear condensation or chromatin fragmentation (without nuclear morphological changes) were identified as apoptotic cells. Results were expressed as TUNEL-positive index (number of TUNEL-positive cells per number of total cells quantified from DAPI-positive counts).

Caspase-3/7 activity assay

The enzymatic activation of effector caspase-3/7 was evaluated in HCC cells following different treatments by using Caspase-Glo® *3/7* assay (Promega) accordingly to manufacturers' instructions. Experiments were performed in quadruplicate.

Bromodeoxyuridine incorporation assay

Proliferative activity was assessed by 5-bromo-2-deoxyuridine (BrdU) labeling (Roche, Mannheim, Germany) following the manufacturer's instructions. The absorbance obtained in the ELISA was measured at 450 nm against a background control at 690 nm (Multiskan Ascent, Leuven, Belgium). Experiments were performed in quadruplicate.

Total RNA extraction

Total RNA was extracted from all samples using the RNeasy Mini Kit (Qiagen, Westburg BV, The Netherlands) with on-column DNAse treatment (Qiagen). Needle homogenization was performed. The purity and quantity of total RNA was assessed using spectrophotometry (Nanodrop; Thermo Scientific, Wilmington, USA). The ratio of absorption at 260 and 280 nm was used to define RNA purity; samples with a 260:280 ratio between 1.8 and 2.0 were accepted.

Quantitative real-time PCR

One microgram of total RNA was converted to single strand cDNA by reverse transcription (iScript, BioRad, California, USA) with oligo (dT) and random priming. The cDNA was diluted 1/10 and used for real-time quantification using SYBR Green (Sensimix, Bioline Reagents Ltd, London, UK) and 250 mM of each primer. A two-step program was run on a LightCyclerR 480 (Roche). Cycling conditions were 95°C for 10 minutes and 45 cycles of 95°C for 10 seconds followed by 1 cycle of 60°C for 1 minute. Melting curve analysis confirmed primer specificities. All reactions were performed in duplicate. Fold change expression was calculated using the ΔΔCT method instructed by Applied Biosystems. Firstly normalization of the threshold cycle (CT) values

of the target gene was done with the CT of GAPDH in the same samples (ΔCT = CT target – CT GAPDH). It was again normalized with the control (ΔΔCT = ΔCT – ΔCT control) and fold change was calculated ($2^{-\Delta\Delta CT}$). The PCR-efficiency of each primer pair was calculated using a standard curve of reference cDNA. Amplification efficiency was determined using the formula $10^{-1/slope}$. The primer set sequences are listed in Table S3.

Western blotting

Total protein extract was obtained by dissolving in RIPA buffer (1× PBS, 1% NP-40, 0.5% sodium deoxycholate, 0.1% SDS and 1× complete protease inhibitors (Roche Diagnostics)). The total protein yield was determined using Bradford reagent (Biorad). 25–50 µg of proteins were loaded on and fractionated by SDS-PAGE. The proteins were transferred to PVDF membrane which was subsequently blocked and incubated with specific antibodies (Table S4) in 5% of non-fat milk followed by horse radish peroxidase-conjugated secondary antibodies. First, the effect of proteasome inhibition on the expression of Tubulin, β-actin and GAPDH was assessed. Tubulin exhibited the most stable expression and was used as loading control. ECL detection reagent (Amersham Life Science, New Jersey, USA) was used to visualize the specific proteins. The densitometric analysis was performed using ImageJ [1].

REFERENCES

1. Schneider CA, Rasband WS, Eliceiri KW. NIH Image to ImageJ: 25 years of image analysis. Nat Methods. 2012; 9:671–5.
2. Chou T-C. Drug combination studies and their synergy quantification using the Chou-Talalay method. Cancer Res. 2010; 70:440–6. doi:10.1158/0008-5472.CAN-09-1947.

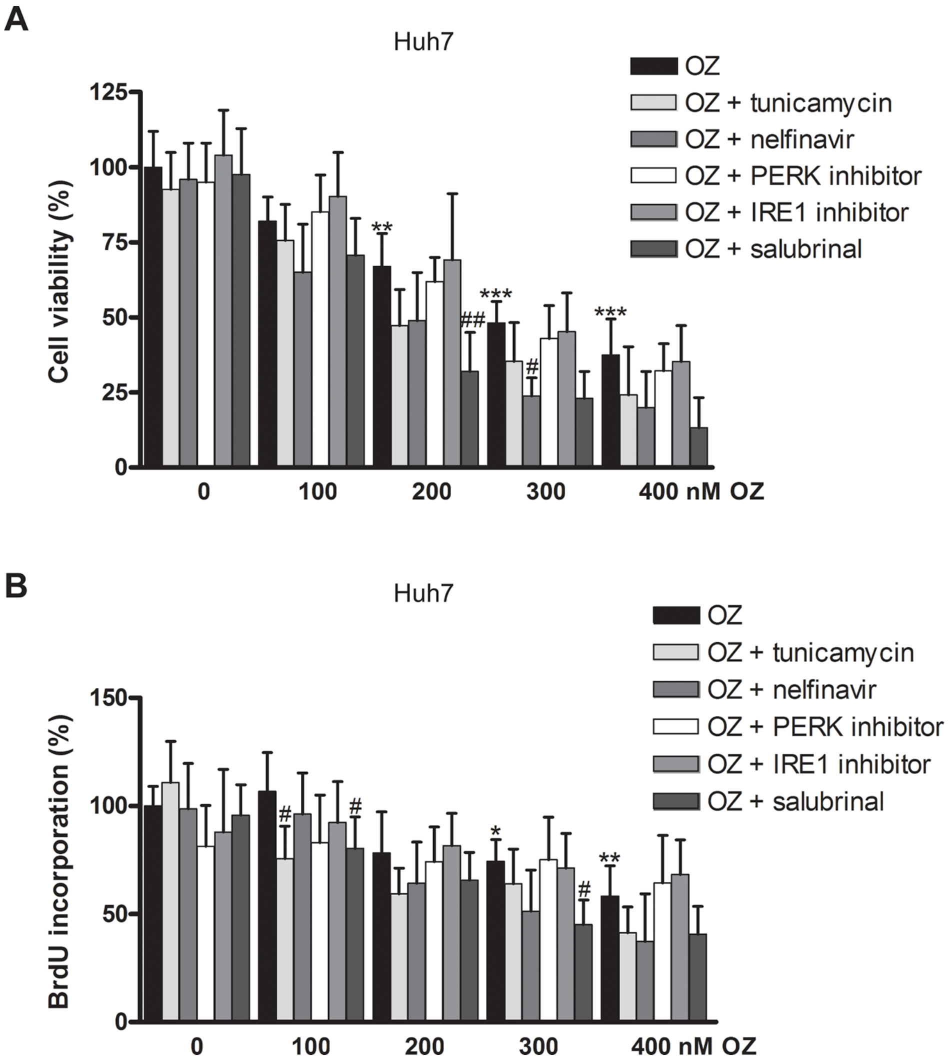

Supplementary Figure S1: Growth-inhibitory effects of oprozomib in Huh7 cells. (**A**) Assessment of cell viability by MTT assay. (**B**) Cell proliferation rate as assessed by BrdU incorporation in Huh7 cells. One-way A7NOVA was applied for statistical analysis. $^{*}p < 0.05$, $^{**}p < 0.01$, $^{***}p < 0.001$ compared to oprozomib 0 nM; $^{\#}p < 0.05$, $^{\#\#}p < 0.01$, $^{\#\#\#}p < 0.001$ compared to respective concentration of oprozomib alone. OZ: oprozomib. Results are representative of 2 independent experiments.

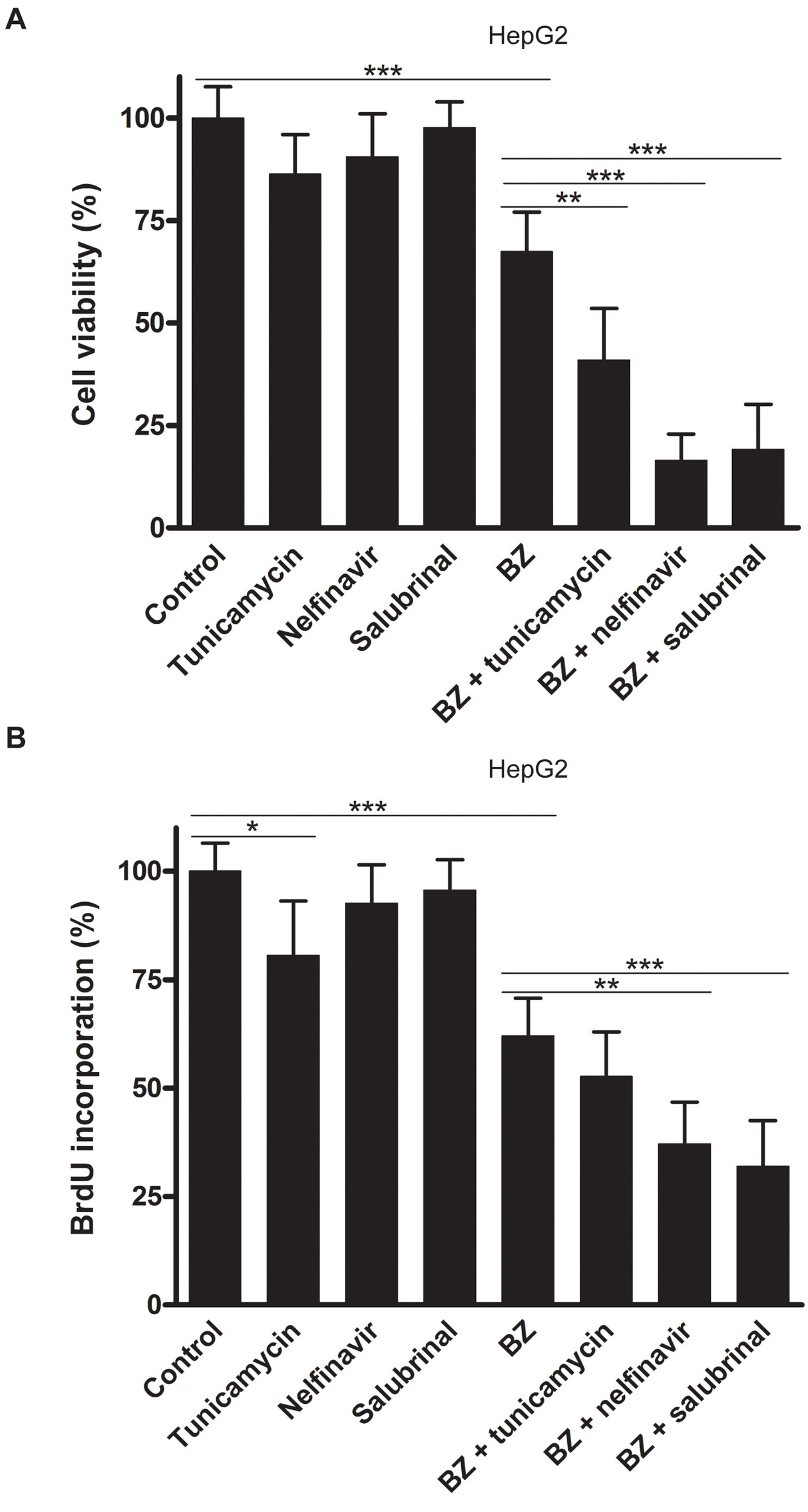

Supplementary Figure S2: Bortezomib and ER stress modulation in HepG2 cells. (**A**) Cell viability as assessed by MTT assay. (**B**) Cell proliferation rate as assessed by BrdU incorporation. *p < 0.05, **p < 0.01, ***p < 0.001. BZ: bortezomib 25 nM. Results are representative of 3 independent experiments.

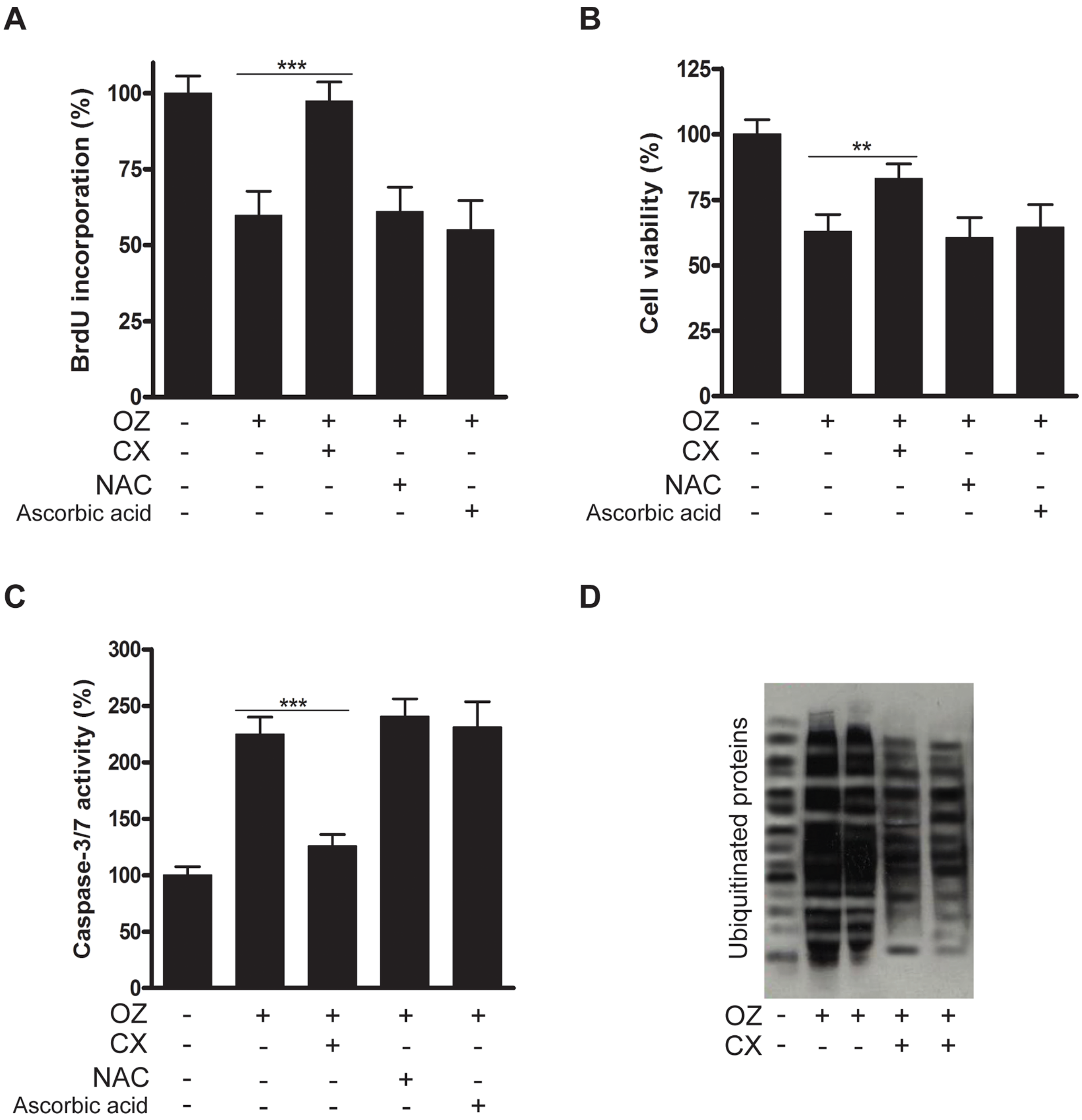

Supplementary Figure S3: Modulation of proteotoxic, not oxidative, stress affects antitumor activity of oprozomib in HepG2 cells. (**A**) Cell proliferation rate. (**B**) Cell viability. (**C**) Caspase-3/7 activity. Values represent the mean ± SD. *$p < 0.05$, **$p < 0.01$, ***$p < 0.001$ compared to oprozomib alone. Results are representative of 2 independent experiments. NAC: N-acetyl-L-cysteine. (**D**) Immunoblotting of poly- and monoubiquitinylated proteins. OZ: oprozomib 400 nM. CX: cycloheximide.

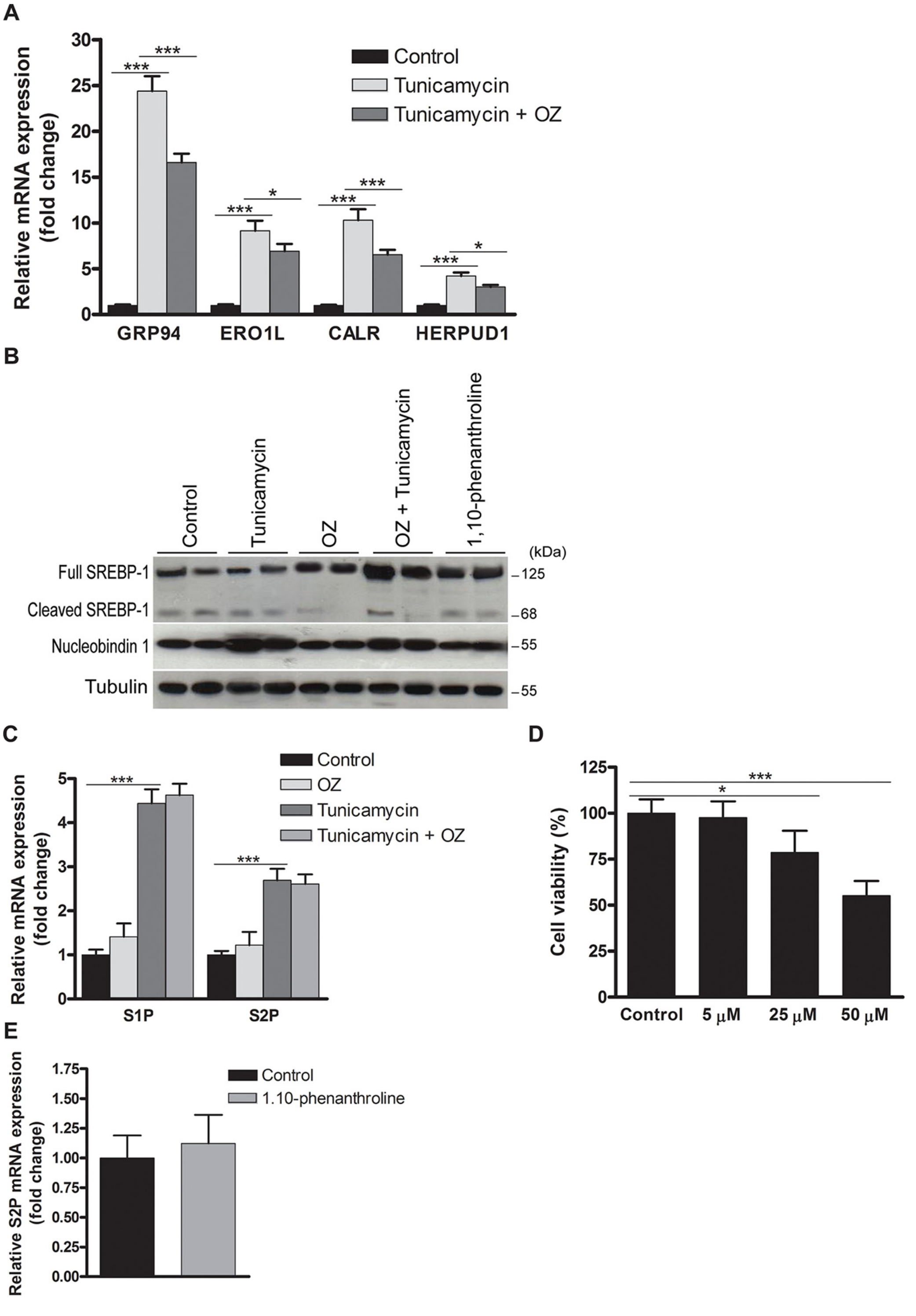

Supplementary Figure S4: OZ inhibits cytoprotective ATF6 activation in HepG2 cells. (**A**) Quantitative real-time PCR analysis of the ATF6 targets GRP94, ERO1L, CALR and HERPUD1. (**B**) Western blotting for RIP-dependent SREBP-1 cleavage and the ATF6 repressor Nucleobindin 1. RIP inhibitor 1,10-phenanthroline was applied at a dose of 25 μM. (**C**) Quantitative real-time PCR analysis of S1P and S2P. OZ: oprozomib 400 nM. (**D**) Effect of 5 to 50 μM 1,10-phenanthroline on the MTT cell viability of HepG2 cells. Results are representative of 2 independent experiments. (**E**) Effect of incubation with 25 μM 1,10-phenanthroline for 48 h on the S2P transcription in HepG2 cells. *$p < 0.05$, **$p < 0.01$, ***$p < 0.001$.

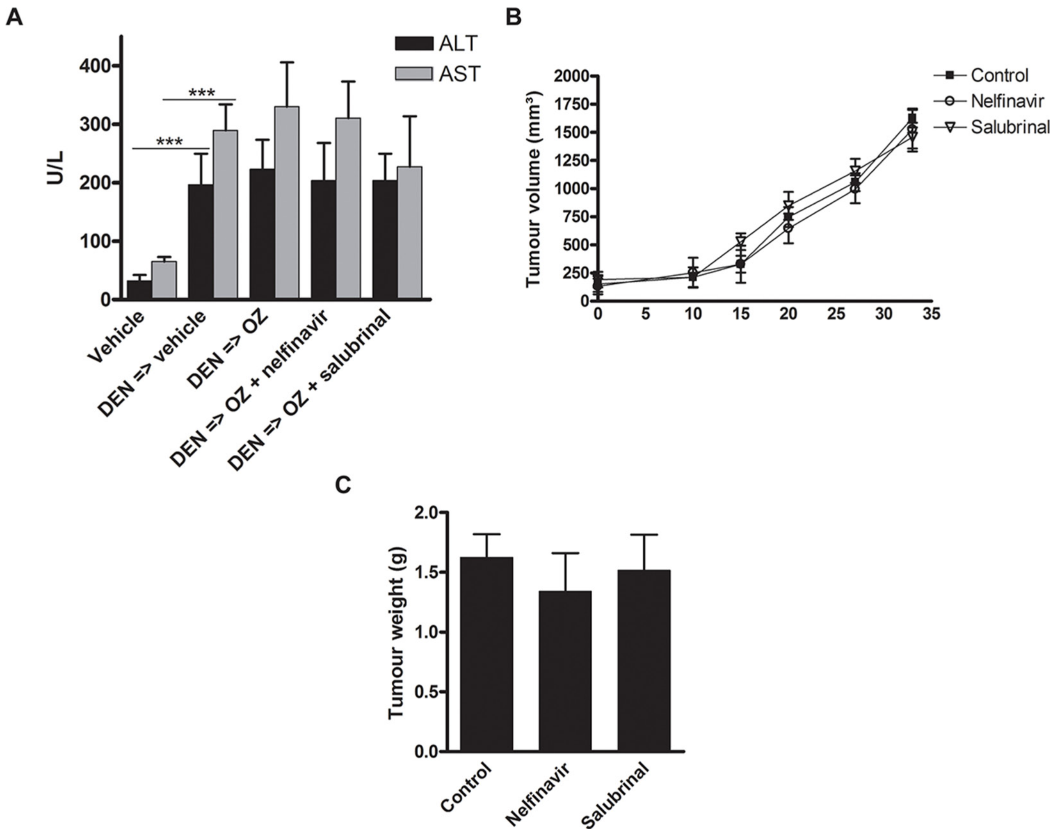

Supplementary Figure S5: Effect of nelfinavir and salubrinal on the serum levels of liver enzymes in the DEN-induced model and on HepG2 xenograft growth. (**A**) Liver damage was assessed by measuring ALT and AST levels in the serum of surviving mice after the indicated treatments. Values represent the mean ± SD ($n = 7$). ***$p < 0.001$. (**B**) Evolution of tumor volume in mice bearing HepG2-derived xenograft tumors. (**C**) Final tumor weights. Values represent the mean ± SD of the tumor volumes of each group.

Supplementary Table S1: IC_{50} and mean combination index values of HCC cells determined by MTT assay

Cells	Treatment	IC_{50} (nM of OZ)*	Percent change compared to OZ monotherapy	Mean combination index (CI)**
HepG2	OZ	307.32	0%	N/A
	OZ + tunicamycin	229.41	–25.35%	0.71
	OZ + nelfinavir	195.09	–36.52%	0.68
	OZ + PERK inhibitor	278.13	–9.50%	0.98
	OZ + IRE1 inhibitor	301.65	–1.84%	1.02
	OZ + salubrinal	187.51	–38.99%	0.60
Huh7	OZ	305.54	0%	N/A
	OZ + tunicamycin	228.27	–25.29%	0.72
	OZ + nelfinavir	203.93	–33.26%	0.69
	OZ + PERK inhibitor	280.31	–8.26%	0.99
	OZ + IRE1 inhibitor	303.85	–0.55%	1.06
	OZ + salubrinal	193.21	–36.76%	0.63

*The IC50 values were obtained using the Bliss method. **The combination effect was assessed by the method described by Chou and Talalay [2] as well as the do's and don'ts in drug combination studies, in terms of experimental design, data acquisition, data interpretation, and computerized simulation. The Chou-Talalay method for drug combination is based on the

median-effect equation, derived from the mass-action law principle, which is the unified theory that provides the common link between single entity and multiple entities, and first order and higher order dynamics. This general equation encompasses the Michaelis-Menten, Hill, Henderson-Hasselbalch, and Scatchard equations in biochemistry and biophysics. The resulting combination index (CI using CompuSyn software (ComboSyn Inc., Paramus, NJ) and a mean combination index, resulting from separate experiments at multiple effect levels, was calculated. A CI < 1 indicates a synergistic interaction, CI = 1 is additive and CI >1 is antagonistic. N/A, not applicable. All compounds added to the dilution series of oprozomib (OZ) were applied at noncytotoxic doses.

Supplementary Table S2: Determination of the mean combination index (CI) of oprozomib (OZ) in combination with nelfinavir in HepG2 cells incubated for 48 hours

Combination	OZ (nM)	Nelfinavir (µM)	FA combination*	CI**
1	100	10	0.35	0.59
2	200	10	0.51	0.70
3	300	10	0.74	0.54
4	400	10	0.80	0.58
5	100	20	0.39	0.63
6	200	20	0.46	0.87
7	300	20	0.53	0.98
8	400	20	0.77	0.67
9	100	30	0.41	0.70
10	200	30	0.62	0.66
11	300	30	0.69	0.70
12	400	30	0.81	0.59
13	100	40	0.44	0.72
14	200	40	0.61	0.69
15	300	40	0.70	0.74
16	400	40	0.89	0.46
				Mean: 0.68

*FA combination: fraction affected values (e.g., FA combination = 0.5 corresponds to 50% decrease in viability).
**The combination effect was assessed by the method described by Chou and Talalay [2] as well as the do's and don'ts in drug combination studies, in terms of experimental design, data acquisition, data interpretation, and computerized simulation. The Chou-Talalay method for drug combination is based on the median-effect equation, derived from the mass-action law principle, which is the unified theory that provides the common link between single entity and multiple entities, and first order and higher order dynamics. This general equation encompasses the Michaelis-Menten, Hill, Henderson-Hasselbalch, and Scatchard equations in biochemistry and biophysics. The resulting combination index (CI using CompuSyn software (ComboSyn Inc., Paramus, NJ).

Supplemantary Table S3: Primers used for qRT-PCR experiments

Gene symbol	Reference sequence	Species	Forward primer	Reverse primer	Efficiency	R^2
GAPDH	NM_001256799.1	Homo sapiens	TGCACCACCAACT-GCTTA GC	GGCATGGACTGTGGT CATGAG	91.9	0.99
ATF4	NM_001675.2	Homo sapiens	GACCACGTTGGAT-GACACTTG	GGGAAGAGGTTGTA-AGAAGGTG	97.6	0.99
CHOP	NM_001195053.1	Homo sapiens	AAGGCACTGAGCG-TATCATGT	TGAAGATA-CACTTCCTTCTT-GAACA	105.2	0.99
XBP1u	NM_001079539.1	Homo sapiens	AGACAGC-GCTTGGGGATGGAT	CCTGCTGCAGAGGT-GCACGTAG	115.0	0.99
XBP1s	NM_001079539.1	Homo sapiens	AGACAGC-GCTTGGGGATGGAT	CCTGCACCTGCTGC-GGACTC	110.1	0.99
PDIA4	NM_004911.4	Homo sapiens	TCCCATTCCTGTTGC-CAAGAT	GCCCTCGTAGTCTA-CAGCCT	99.8	0.99
GRP78	NM_005347.4	Homo sapiens	GGGAACGTCT-GATTGGCGAT	CGTCAAAGACCGT-GTTCTCG	106.1	0.99
GADD34	NM_014330.3	Homo sapiens	TCCTCTG-GCAATCCCCCATA	GGAACTGCTG-GTTTTCAGCC	109.0	0.99
ERO1L	NM_014584.1	Homo sapiens	GCCAGGTTAGTGGT-TACTTGG	GGCCTCTTCAGGTT-TACCTTGT	108.1	0.99
GRP94	NM_003299.2	Homo sapiens	GCTGACGAT-GAAGTTGATGTGG	CATCCGTCCTT-GATCCTTCTCTA	103.2	0.98
HER-PUD1	NM_001010989.2	Homo sapiens	TTCCAAAGCAG-GAAAAACGGC	GCAGGCTCCTCT-GTGGATTC	93.7	0.95
CALR	NM_004343.3	Homo sapiens	CCTGCCGTC-TACTTCAAGGAG	GAACTTGCCG-GAACTGAGAAC	94.5	0.99
S1P	NM_003791.3	Homo sapiens	ACCTCGAAACAATC-CATCCAGT	ACTTGAGGGAAC-GAAAGACTTTT	99.7	0.99
S2P	NM_015884.3	Homo sapiens	TGGACTGTCGTC-TACCTGACC	AGCAGTTTGCCATCT-TATGTGG	97.8	0.97
ERDJ4	NM_012328.2	Homo sapiens	GGTGTGCCAAAATC-GGCATC	GCACTGTGTC-CAAGTGTATCATA	100.3	0.98
Gapdh	NM_008084.2	Mus muscu-lus	GCCGGCTCAGT-GAGACAAG	TGGCACCTTCAG-CAACAATG	95.1	0.99
Chop	NM_007837.3	Mus muscu-lus	AGCGCAACATGA-CAGTGAAG	GTGTAATTC-CAGGGGGAGGT	101.2	0.99
Pdia4	NM_009787.2	Mus muscu-lus	ACGAGACCCCGGC-GTTCGGA	TGGCACTTTGAG-GAGGTGAGCC	92.6	0.99
Grp78	NM_001163434.1	Mus muscu-lus	TGCCGAGCTAAATTA-CACATTG	CCTTGTGGAGGGAT-GTACAGA	107.1	0.99

The PCR-efficiency of each primer pair was calculated using a standard curve of reference cDNA. Amplification efficiency R^2 was determined using the formula $10^{-1/\text{slope}}$.

Supplementary Table S4: Characteristics of the antibodies used in the study

Antigen	Antibody isotype, clone	Company	Cat no.
ATF4	Rabbit polyclonal IgG	Santa Cruz	sc-200
eIF2α	Rabbit polyclonal IgG	Cell Signaling	9721
Phospho-eIF2α	Rabbit monoclonal IgG, 119A11	Cell Signaling	3597
CHOP	Mouse monoclonal IgG2a, L63F7	Cell Signaling	2895
GADD34	Rabbit polyclonal IgG, H193	Santa Cruz	sc-8327
GRP78	Rabbit monoclonal IgG, C50B12	Cell Signaling	3177
PDIA4	Rabbit polyclonal IgG	Cell Signaling	2798
ATF6	Mouse monoclonal IgG, 70B1413.1	Imgenex	img-273
SREBP-1	Mouse monoclonal IgG1, 2A4	Abcam	ab3259
Nucleobindin 1	Rabbit polyclonal IgG	Abcam	ab26093
Mono- and polyubiquitinylated conjugates	Mouse monoclonal IgG1, FK2	Enzo Life Sciences	bml-pw8810
B-tubulin	Rabbit polyclonal IgG	Abcam	ab6046
Actin	Rabbit polyclonal IgG	Abcam	ab5694-100

The specificity, isotype, clone number, and catalog number of the antibodies are indicated if provided.
